# Supplementary figures and images for: Comparison of Hydrogels for the Development of Well-Defined 3D Cancer Models of Breast Cancer and Melanoma
Source: Cancers (Basel). 2020 Aug 17;12(8):2320. doi: 10.3390/cancers12082320 (PMC7465483; doi:10.3390/cancers12082320)

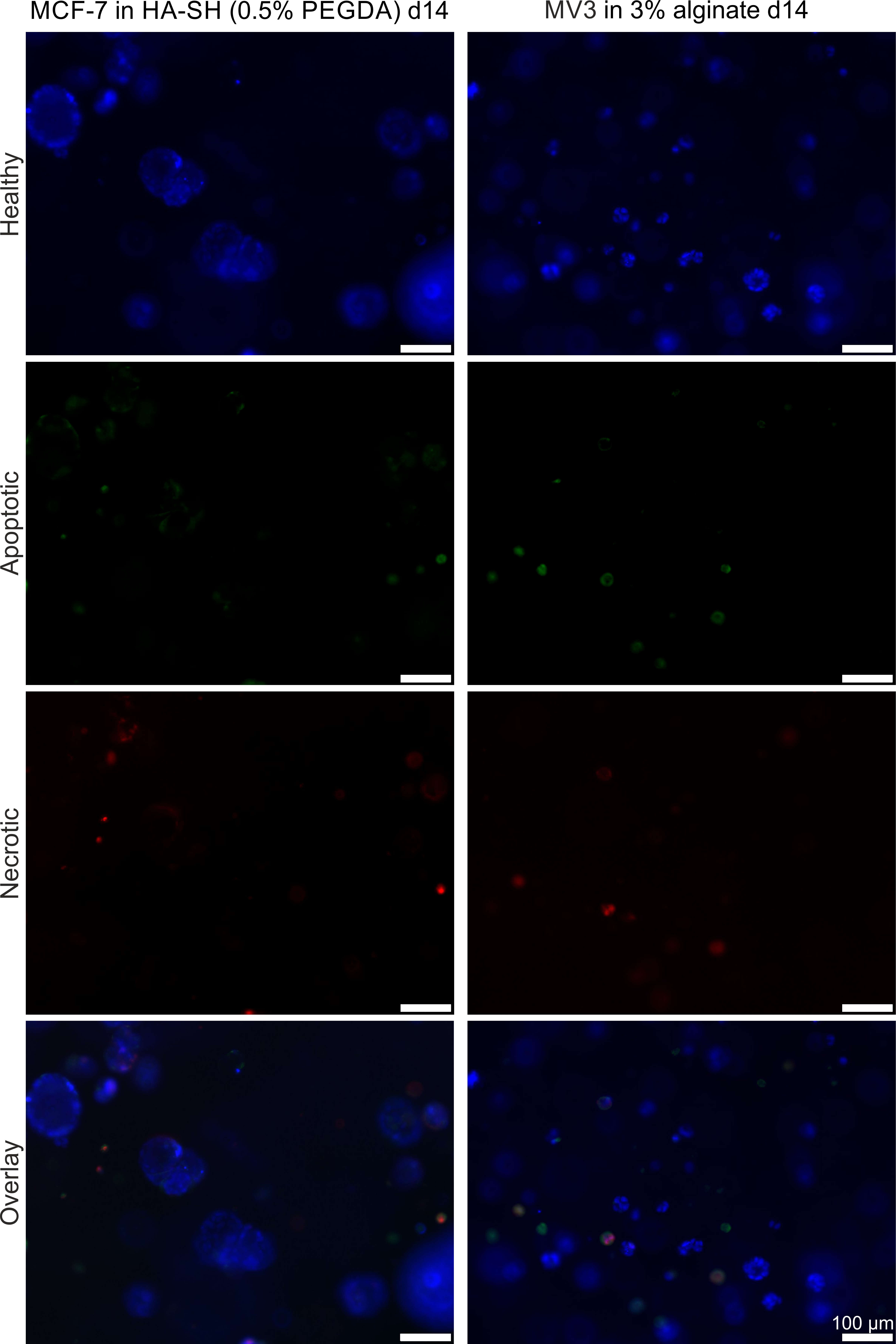

Supplement: Supplementary file 1 [file cancers-12-02320-s001.zip › cancers-878440.jpg]
